# Robot- and Technology-Boosting Neuroplasticity-Dependent Motor-Cognitive Functional Recovery: Looking towards the Future of Neurorehabilitation

**DOI:** 10.3390/brainsci13121687

**Published:** 2023-12-07

**Authors:** Giovanni Morone, Marco Iosa, Rocco Salvatore Calabrò, Antonio Cerasa, Stefano Paolucci, Gabriella Antonucci, Irene Ciancarelli

**Affiliations:** 1 Department of Life, Health and Environmental Sciences, University of L’Aquila, 67100 L’Aquila, Italy; giovanni.morone@univaq.it (G.M.); irene.ciancarelli@univaq.it (I.C.); 2 San Raffaele Institute of Sulmona, Viale dell’Agricoltura, 67039 Sulmona, Italy; 3 Department of Psychology, Sapienza University of Rome, 00185 Rome, Italy; gabriella.antonucci@uniroma1.it; 4 Santa Lucia Foundation, Scientific Institute for Research, Hospitalization and Health Care (IRCCS), 00179 Rome, Italy; s.paolucci@hsantalucia.it; 5 Neurorehabilitation Unit, IRCCS Neurolesi Center “Bonino-Pulejo”, 98124 Messina, Italy; roccos.calabro@irccsme.it; 6 S’Anna Institute, 88900 Crotone, Italy; antonio.cerasa@irib.cnr.it; 7 Institute for Biomedical Research and Innovation (IRIB), National Research Council of Italy (CNR), 98164 Messina, Italy; 8 Translational Pharmacology, Department of Pharmacy, Health and Nutritional Sciences, University of Calabria, 87036 Rende, Italy

The sequelae of neurological disorders are the leading causes of disability in all industrialized countries. Conventional rehabilitation usually allows a small proportion of patients suffering from neurological disabilities to completely recover independent walking or functional grasping, and other activities of daily living [1]. For these reasons, an increasing number of research studies and randomized clinical trials are pursuing the use of new robots and technologies to improve the efficacy of rehabilitation [2,3,4,5,6]. They have become more usable and widespread every year, thanks to new principles of neuroscience translated into clinical practice through technological innovations. However, despite their diffusion in neurorehabilitation, many questions remain unanswered.

In particular, the disputes about their efficacy, together with the high purchase cost for most of these devices [7,8], the absence of clear and univocal guidelines for better dosages to use and parameter values to set, and the somewhat diffuse skepticism of some members of the rehabilitation teams, may limit their use in clinical settings.

Finally, most of the available studies and clinical indications focus on stroke and multiple sclerosis, even though robots might be beneficial for other pathologies, such as Parkinson’s disease, traumatic brain injuries, spinal cord injuries, and other brain degenerative diseases [9,10,11].

This Special Issue aims to provide an overview of the use of new technologies in the neurorehabilitation of people with motor and cognitive disabilities stemming from central nervous system diseases.

In general, studies address the effectiveness of therapy versus conventional therapy, while in daily clinical practice, the clinician must choose the suitable type of neurorehabilitation assisted by a specific robot for each specific patient [12]. For this reason, in recent years, the ideal type of robot and related cognitive stimulation for patients with specific characteristics has been investigated in line with personalized medicine [13,14]. Therefore, a Special Issue that addresses the use of technologies in multiple types of patients and for different objectives can represent a step forward in increasing our knowledge on the topic and arriving at a competent and skillful use of robotics and technology in neurorehabilitation clinics.

In this Special Issue, two systematic reviews investigated the efficacy of robot-assisted gait training (RAGT) on balance recovery using overground exoskeletons (Lorusso M et al.) and of many different robotic devices including overground exoskeletons, grounded exoskeletons, and end-effectors (Loro A et al.). Xie et al. investigated the optimal intervention timing of RAGT, with two protocols allowing us to better understand the clinical effects of robot-assisted therapy for arm function recovery (Pournajaf S. et al.) and for walking recovery (Kolářová, B et al.) by means of two distinct, well-planned, and methodologically rigorous randomized controlled trials. Finally, a feasibility study investigated the use of an intelligent algorithm based on an assist-as-needed controller in RAGT, which was conducted by Laszlo C. and co-authors.

But, robots are not the only emerging technology in neurorehabilitation. In two pilot studies by De Luca R and a systematic review, the potential of virtual reality (VR) in neurorehabilitation was examined. The first study looked at how executive functioning and coping mechanisms in traumatic brain injury patients might be improved with VR-based cognitive rehabilitation training, and the second examined how traumatic brain injury patients’ attention processes might be affected by non-immersive VR training. The systematic review was conducted by Martino Cinnera et al. and explored the efficacy of VR in patients with unilateral spatial neglect due to stroke. Regarding the gender differences in subjects affected by traumatic brain injuries, Bruschetta R et al. demonstrated that females who underwent VR training showed better cognitive recovery. 

The technologies aiming to modulate neuroplasticity and thus improve the function for reducing pain should be combined to allow for an improvement in the clinical effects. This is the case of two studies in this Special Issue. In one study, De Luca R et al. combined robotic verticalization and music therapy in chronic disorders of consciousness, and in another study, Calabrò et al. combined transcranial magnetic stimulation and muscle vibration for women with chronic pelvic pain.

Sato M. et al. investigated the different contributions of the frequency and the duration of PES, peripheral sensory nerve electrical stimulation, on the excitability of the primary motor cortex. Facciorusso S. et al. performed a bibliometric analysis of research trends regarding sensor-based rehabilitation in neurological diseases.

Finally, a study conducted by Varalta V. et al. demonstrated an improvement in global cognitive status and in attention functions when subjects affected by Parkinson’s disease were treated with an upper limb motor protocol, underlining the strong interconnections that exist between motor and cognitive functions for the upper limbs.

There is no doubt that robots and technologies are changing clinicians’ ways of thinking in rehabilitation, and even if they are not the definitive solution for improving plasticity-dependent functional recovery, they will certainly play a fundamental role in improving the efficacy of neurorehabilitation.
